# Association of Type 2 Diabetes Mellitus With Dietary Vitamin B12 Intake in Individuals With Different Fat Mass and Obesity‐Associated (FTO) Genotypes

**DOI:** 10.1002/fsn3.71783

**Published:** 2026-06-17

**Authors:** Elham Nikoei Foshtomi, Atefeh Tahavorgar, Ali Shamsi‐Goushki, Mahsa Vahdat, Pegah Samani, Maryam Shojaei, Mohammadtaghi Ghorbani Hesari, Ali Moradi, Morteza Abdollahi, Anahita Houshyar Rad, Saeid Doaei, Marjan Ajami, Maryam Gholamalizadeh

**Affiliations:** ^1^ Faculty of Sport Sciences and Physical Education University of A Coruña A Coruña Spain; ^2^ School of Nutritional Sciences and Dietetics Tehran University of Medical Sciences Tehran Iran; ^3^ Department of Nutrition, School of Medicine Mashhad University of Medical Sciences Mashhad Iran; ^4^ Aboozar Children's Medical Center Ahvaz Jundishapur University of Medical Sciences Ahvaz Iran; ^5^ Nutrition and Food Security Research Center Shahid Sadoughi University of Medical Science Yazd Iran; ^6^ Central Research Laboratory, School of Medicine Shiraz University of Medical Sciences Shiraz Iran; ^7^ Mashhad University of Medical Sciences Mashhad Iran; ^8^ School of Medicine Tehran University of Medical Sciences Tehran Iran; ^9^ Social Determinants of Health Research Center Shahid Beheshti University of Medical Sciences Tehran Iran; ^10^ National Nutrition and Food Technology Research Institute and Faculty of Nutrition and Food Technology Shahid Beheshti University of Medical Sciences Tehran Iran; ^11^ Department of Community Nutrition, Faculty of Nutrition Sciences and Food Technology, National Nutrition and Food Technology Research Institute Shahid Beheshti University of Medical Sciences Tehran Iran; ^12^ Unit of Nutrition and Cancer, Cancer Epidemiology Research Program Catalan Institute of Oncology, Bellvitge Biomedical Research Institute (IDIBELL), L’Hospitalet de Llobregat Barcelona Spain; ^13^ Department of Food and Nutrition Policy and Planning Research, Faculty of Nutrition Sciences and Food Technology, National Nutrition and Food Technology Research Institute Shahid Beheshti University of Medical Sciences Tehran Iran; ^14^ Faculty of Nutrition Sciences and Food Technology, National Nutrition and Food Technology Research Institute Shahid Beheshti University of Medical Sciences Tehran Iran

**Keywords:** FTO gene, rs9939609 polymorphism, type 2 diabetes mellitus, vitamin B12

## Abstract

Type 2 diabetes mellitus (T2DM) is a multifactorial disease influenced by genetic and environmental factors. The fat mass and obesity‐associated (FTO) gene has been implicated in obesity and metabolic disorders, while vitamin B12 plays a role in glucose metabolism and insulin sensitivity. This study aimed to assess the interaction between dietary vitamin B12 intake and FTO genotypes in relation to T2DM risk. A case–control study was conducted on 400 participants (179 with T2DM and 174 controls) aged 35–70 years. Dietary intake was assessed using a validated food frequency questionnaire, and genotyping of the FTO rs9939609 polymorphism was performed using the tetra‐primer amplification refractory mutation system polymerase chain reaction (Tetra‐ARMS PCR). Logistic regression models were applied to evaluate the association between dietary vitamin B12 intake and T2DM risk, stratified by FTO genotype (TT versus AA/AT). Vitamin B12 intake was significantly higher in participants with the TT genotype of the FTO gene in the control group compared to participants with the TT genotype in the case group (5.44 ± 2.37 vs. 4.03 ± 2.13 μg/day, *p* = 0.004). Vitamin B12 showed a significant inverse association with T2DM (OR = 0.778, 95% CI 0.636–0.952) in the TT group, whereas no significant associations were observed for the AA/AT group. Higher dietary vitamin B12 intake associated with lower odds of T2DM in individuals with the TT genotype of the FTO rs9939609 polymorphism, suggesting a gene–nutrient interaction that may inform precision nutrition strategies for diabetes prevention.

## Graphical abstract

Higher dietary vitamin B12 intake was inversely associated with the odds of type 2 diabetes mellitus among individuals carrying the TT genotype of the FTO rs9939609 polymorphism. These findings highlight the potential role of precision nutrition strategies in diabetes prevention.

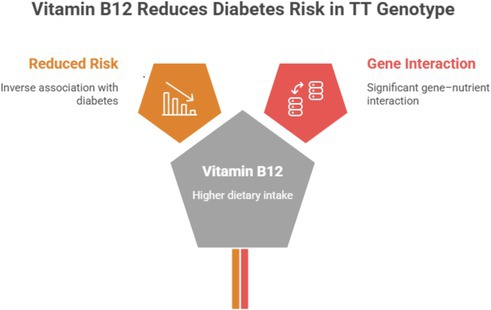

## 1 Introduction

Type 2 diabetes mellitus (T2DM) is characterized by varying degrees of pancreatic beta cell dysfunction, hyperglycemia, and insulin resistance (Abel et al. 2024). The global prevalence of T2DM has increased dramatically over the past decades due to changes in lifestyle, urbanization, and population aging (Yu et al. 2025). Recent global estimates indicate that approximately 589 million adults (aged 20–79) are living with diabetes, a number projected to exceed 850 million by 2050, if current trends persist (Genitsaridi et al. 2026). Diabetes is associated with macrovascular complications, including coronary heart disease, stroke, and peripheral vascular disease, as well as microvascular complications, which include end‐stage renal disease (ESRD), retinopathy, and neuropathy (Diabetes Care 2011; Gkrinia and Belančić 2025).

The pathogenesis of T2DM is multifactorial, arising from complex interactions between genetic predisposition, environmental exposures, and behavioral factors. Obesity, physical inactivity, unhealthy dietary patterns, and aging are well‐established risk factors of T2DM (Doaei, Kalantari, Mohammadi, et al. 2019; Abdallah and Mihailescu 2023). Among the genetic determinants of obesity and metabolic disorders, the fat mass and obesity‐associated (FTO) gene has been considered one of the most consistently replicated susceptibility loci (Doaei, Kalantari, Keshavarz Mohammadi, et al. 2019; Kalantari et al. 2018). FTO gene is strongly associated with increased body mass index (BMI), adiposity, and risk of T2DM (McCarthy 2010; Doaei, Kalantari, Keshavarz Mohammadi, et al. 2019). Recent evidence highlights that FTO plays a central regulatory role in metabolic pathways through its effects on RNA epigenetic modifications, influencing energy balance and fat accumulation (Benak et al. 2024). Moreover, emerging studies suggest that dysregulation of FTO demethylase activity contributes to the development of obesity‐related metabolic disorders, further reinforcing its link to diabetes susceptibility (Somala et al. 2025). Along with genetic susceptibility, lifestyle modification including dietary intake and physical activity remains a cornerstone of T2DM prevention and management (Ojo 2019; Doaei, Kalantari, Izadi, et al. 2019). More recently, vitamin B12 (cobalamin) has attracted attention for its important role in T2DM pathophysiology (Madhura et al. 2019). Vitamin B12 plays a key role in homocysteine and methylmalonic acid (MMA) metabolism (van de Lagemaat et al. 2019), and deficiency has been associated with increased oxidative stress, impaired methylation capacity, and insulin resistance (Boachie et al. 2021; Fernández‐Cao et al. 2019). Emerging evidence suggests that low vitamin B12 status may contribute to impaired glucose metabolism, increased oxidative stress, and altered mitochondrial function, all of which are implicated in the development of insulin resistance (Capitão et al. 2017; Liu et al. 2020; Knight et al. 2015). Furthermore, another study has reported that long‐term metformin therapy can significantly reduce circulating vitamin B12 levels, highlighting the clinical relevance of monitoring B12 status in diabetic populations (Al Quran et al. 2023).

Because FTO encodes an epigenetic demethylase, and vitamin B12 is essential for one‐carbon metabolism and methylation processes, its effect on metabolic outcomes and type 2 diabetes risk may differ in individuals carrying FTO genetic variants compared with non‐carriers. Despite growing evidence supporting independent roles of genetics and dietary interventions in T2DM, limited studies have assessed the interactions between T2DM, the FTO gene, and vitamin B12. Therefore, the present study aimed to investigate the association between dietary cobalamin intake and T2DM across different FTO genotypes, providing insight into potential gene–nutrient interactions involved in the development of type 2 diabetes.

## 2 Methods

### 2.1 Participants

**FIGURE 1:**
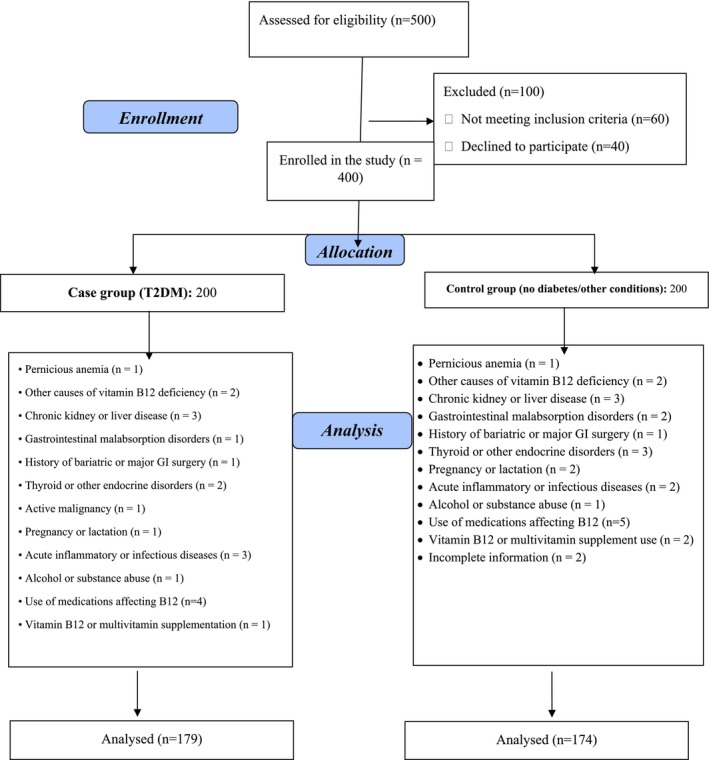
Flowchart of the study participants.

This case–control study initially included 400 women, comprising 200 participants with T2DM and 200 women without diabetes or any other known medical conditions. All participants were between 35 and 70 years of age and were randomly selected from women attending Shohadaye Tajrish Hospital in Tehran, Iran. Participants were recruited between March 2022 and March 2024. Control subjects were selected from the same hospital among women who visited the facility for routine health examinations. The inclusion criteria included women aged 35–70 years, a confirmed diagnosis of T2DM in the case group, a written informed consent form, and absence of food restrictions due to special diets or food sensitivities. Type 2 diabetes mellitus (T2DM) was defined as at least one of the following: fasting plasma glucose (FPG) ≥ 126 mg/dL (7.0 mmol/L), 2‐h plasma glucose ≥ 200 mg/dL (11.1 mmol/L) following a 75‐g oral glucose tolerance test (OGTT), or glycated hemoglobin (HbA1c) ≥ 6.5%. Participants with pre‐diabetes, defined according to ADA criteria (FPG 100–125 mg/dL, 2 h‐OGTT 140–199 mg/dL, or HbA1c 5.7%–6.4%), were not included to ensure a clear distinction between the T2DM and control groups. Participants were excluded if they had conditions known to influence vitamin B12 status or confound its association with diabetes, including pernicious anemia (*n* = 2), other causes of vitamin B12 deficiency (*n* = 4), chronic kidney or liver disease (*n* = 6), gastrointestinal malabsorption disorders (*n* = 3), a history of bariatric or major gastrointestinal surgery (*n* = 2), thyroid or other endocrine disorders (*n* = 5), active malignancy (*n* = 1), pregnancy or lactation (*n* = 3), acute inflammatory or infectious diseases (*n* = 5), or alcohol or substance abuse (*n* = 2). Participants using medications known to affect vitamin B12 levels including proton pump inhibitors or H2‐receptor antagonists, anticonvulsants, or other B12‐modifying drugs (*n* = 9) as well as those who had taken vitamin B12 or multivitamin supplements (*n* = 3) or provided incomplete information (*n* = 2) were also excluded. Of the 400 women enrolled in the study, 47 participants met at least one exclusion criterion and were removed, resulting in a final analytic sample of 353 individuals including 179 individuals with type 2 diabetes and 174 non‐diabetic controls (Figure 1). For subgroup analyses based on FTO genotype, among cases, 58 individuals carried the TT genotype and 121 carried the AT/AA genotypes, whereas among controls, 62 individuals carried the TT genotype and 112 carried the AT/AA genotypes. These genotype‐specific counts were used in all stratified and interaction models.

Information related to the demographic and social status of the participants was collected from the general information questionnaire. To measure weight and height, the SECA 755 mechanical column scale and SECA 204 mobile stadiometer were used, respectively. The BMI of participants was calculated as weight (kg) divided by height squared (m^2^). Physical activity levels were assessed using a validated form of the International Physical Activity Questionnaire (IPAQ) short form, which estimates weekly energy expenditure in MET‐minutes based on self‐reported frequency and duration of walking, moderate, and vigorous activities (Vasheghani‐Farahani et al. 2011).

### 2.2 Dietary Intake

A valid semi‐quantitative food frequency questionnaire (FFQ) comprising 237 items was used to assess dietary intake, including the amount of food consumed and specific preparation methods for certain foods. This questionnaire has been previously validated in Iran (Eghtesad et al. 2023). The data from the FFQ questionnaire were analyzed using Nutritionist IV software to determine the consumption of different types of dietary components, including macronutrients and vitamin B12. Nutrient intakes were calculated for each food item and summed to estimate average daily intake of energy, macronutrients, and vitamin B12.

### 2.3 Determination of the FTO Genotype

Five milliliters blood samples of the participants were collected at the beginning of the study. Genomic deoxyribonucleic acid (DNA) was extracted from blood samples using a GeneAll DNA extraction kit (Incheon, Korea) according to the manufacturer's instructions. The extracted DNA samples were amplified using PCR and Master Mix DNA polymerase (lot no. A180301; Ampliqon, Denmark). The FTO rs9939609 polymorphism was genotyped using the Tetra‐primer Amplification Refractory Mutation System PCR (Tetra‐ARMS PCR), which employs allele‐specific inner primers and outer primers to selectively amplify both the wild‐type and mutant alleles in a single PCR reaction.

### 2.4 Statistical Analysis

Descriptive statistics, including mean and standard deviation, were used to summarize demographic, social, and anthropometric characteristics of the participants, as well as to examine dietary vitamin B12 intake across the groups based on different FTO gene genotypes. Logistic regression was used to assess the relationship between T2DM and dietary intake of vitamin B12 after adjusting for the effect of the confounding variables. Adjusted models controlled for total calorie intake (Model 1); additionally adjusted for age and physical activity (Model 2); and further adjusted for body mass index (BMI) and carbohydrate intake (Model 3). In all analyses, a *p*‐value < 0.05 was considered statistically significant. All statistical procedures were performed using SPSS software, version 22.

## 3 Results

The general characteristics of the participants based on the FTO rs9939609 genotypes are summarized in Table 1. In both FTO genotypes, TT and AA/AT, the controls were older (TT: 53.15 ± 8.53 vs. 47.68 ± 7.67; AA/AT: 53.07 ± 8.73 vs. 47.52 ± 7.64), heavier (TT: 76.21 ± 12.93 vs. 69.22 ± 11.83; AA/AT: 74.73 ± 11.67 vs. 68.60 ± 10.97), and had higher BMI (TT: 31.43 ± 4.40 vs. 28.21 ± 4.34; AA/AT: 30.53 ± 4.11 vs. 28.26 ± 4.12) than the cases. Among participants with the TT genotype, cases had higher physical activity than controls (1.98 ± 1.71 vs. 1.03 ± 1.17; *p* = 0.001). Dietary intake of the participants is presented in Table 2. Among the participants with the TT genotype of FTO, the controls had a higher intake of vitamin B12 (*p* = 0.004) than the cases. This finding was not significant in participants with the AA/AT genotype of FTO. There were no significant differences between the two groups in the calorie, protein, carbohydrate, and total fat intakes in both TT and AA/AT genotypes of FTO.

**TABLE 1:** General characteristics of the participants based on FTO genotypes.

	TT genotype			AA/AT genotypes		
	Controls (*n* = 62)	Cases (*n* = 58)	*p*	Controls (*n* = 112)	Cases (*n* = 121)	*p*
Age (year)	53.15 ± 8.53	47.68 ± 7.67	0.002	53.07 ± 8.73	47.52 ± 7.64	< 0.001
Height (cm)	155.41 ± 4.74	156.49 ± 5.19	0.280	156.303 ± 5.352	155.724 ± 6.086	0.489
Weight (kg)	76.205 ± 12.930	69.215 ± 11.832	0.007	74.730 ± 11.669	68.595 ± 10.969	0.000
BMI (kg/m^2^)	31.433 ± 4.397	28.205 ± 4.342	0.000	30.534 ± 4.110	28.264 ± 4.115	0.000
Physical activity	1.027 ± 1.166	1.984 ± 1.708	0.001	1.493 ± 1.537	1.307 ± 1.458	0.407
FBS	96.50 ± 10.99	136.07 ± 40.07	0.001	96.13 ± 12.29	185.34 ± 85.06	0.001
Having IHD	3 (4.4%)	4 (7.1%)	0.52	5 (4.3%)	25 (20.7%)	0.006

**TABLE 2:** Dietary intake of the participants based on FTO genotypes.

	TT genotype			AA/AT genotypes		
	Controls (*n* = 62)	Cases (*n* = 58)	*p*	Controls (*n* = 112)	Cases (*n* = 121)	*p*
Energy (kcal)	2626.33 ± 391.25	2528.31 ± 521.01	0.314	2615.97 ± 632.51	2551.97 ± 202.27	0.312
Protein (g/d)	86.35 ± 18.64	82.17 ± 23.57	0.348	85.47 ± 32.87	80.21 ± 14.80	0.134
Carbohydrate (g/d)	371.64 ± 71.31	357.63 ± 70.24	0.330	377.67 ± 77.69	365.57 ± 29.02	0.130
Total fat (g/d)	96.07 ± 16.44	92.79 ± 25.27	0.472	92.59 ± 28.39	94.49 ± 15.41	0.551
Vitamin B12 (μg/d)	5.44 ± 2.37	4.03 ± 2.13	0.004	4.74 ± 3.75	3.87 ± 2.45	0.063

The association between T2DM and dietary vitamin B12 intake among carriers of different FTO genotypes is presented in Table 3. Among participants with the TT genotype (*n* = 120), higher dietary intake of vitamin B12 was significantly associated with a lower risk of T2DM across all regression models. In Model 1, higher B12 intake was inversely associated with T2DM (OR = 0.778; 95% CI: 0.636–0.952; *p* = 0.015). This association remained significant after further adjustment for age and physical activity in Model 2 (OR = 0.772; 95% CI: 0.612–0.974; *p* = 0.029), and persisted significant in Model 3 after additional adjustment for BMI and carbohydrate intake (OR = 0.759; 95% CI: 0.559–0.987; *p* = 0.035). In contrast, among carriers of the AA/AT genotypes (*n* = 233), dietary vitamin B12 intake was not significantly associated with T2DM risk in any of the adjusted models (Table 3).

**TABLE 3:** The association of type 2 diabetes with dietary intake of vitamin B12 among carriers of different FTO genotypes.

	TT (*n* = 120)		AA/AT (*n* = 233)	
	OR (95% CI)	*p*	OR (95% CI)	*p*
Model 1	0.778 (0.636–0.952)	0.015	0.911 (0.803–1.034)	0.150
Model 2	0.772 (0.612–0.974)	0.029	0.929 (0.819–1.053)	0.247
Model 3	0.759 (0.559–0.987)	0.035	0.939 (0.815–1.081)	0.379

*Note:* Model 1: Adjusted for calorie intake; Hosmer‐Lemeshow goodness‐of‐fit *X* ^2^ = 13.27, df = 8, *p* = 0.103; Nagelkerke *R* ^2^ = 0.059. Model 2: Further adjusted for age and physical activity; Hosmer‐Lemeshow goodness‐of‐fit *X* ^2^ = 7.55, df = 8, *p* = 0.478; Nagelkerke *R* ^2^ = 0.229. Model 3: Additional adjustment for BMI and carbohydrate intake; Hosmer‐Lemeshow goodness‐of‐fit *X* ^2^ = 7.56, df = 8, *p* = 0.478; Nagelkerke *R* ^2^ = 0.236.

## 4 Discussion

The results of the present study demonstrated a significant inverse association between dietary vitamin B12 intake and the risk of type 2 diabetes among individuals with the TT genotype of the FTO rs9939609 polymorphism. In contrast, no significant association was observed among carriers of the AT/AA genotypes. These findings indicated that genetic variation in the FTO gene might modify the association between vitamin B12 intake and the risk of T2DM.

Several studies reported that vitamin B12 might influence glucose metabolism and insulin sensitivity, as well as oxidative stress processes (van de Lagemaat et al. 2019; Li et al. 2018; Su et al. 2025). Moreover, according to Frayling et al. (Frayling et al. 2007) and Zeggini et al. (Zeggini et al. 2007), FTO polymorphisms have been linked to obesity, a prominent risk factor for T2DM. In addition, the FTO gene is known to have associations with energy balance and metabolism, based on numerous genome‐wide association studies (Khan et al. 2020; Doaei, Mosavi Jarrahi, et al. 2019).

Another study examined the association between vitamin deficiencies and type 2 diabetes (T2DM) among Malaysian adults. Among 9314 participants, 16.9% had T2DM; higher intake of vitamins B6, B9, B12, E, and K was linked to a reduced prevalence of T2DM (Md Isa et al. 2025). These findings were supported by similar studies that concluded deficiency of vitamin B‐complex (B6, B9, and B12) was associated with the risk of T2DM (Mursleen and Riaz 2017; Valdés‐Ramos et al. 2015; Mascolo and Vernì 2020).

On the other hand, several studies have reported null or inconsistent associations between vitamin B12 status and the risk of developing type 2 diabetes, suggesting that the relationship may not be uniform across populations (O'Leary and Samman 2010; Shahjahan et al. 2024; Kibirige and Mwebaze 2013). For example, longitudinal analyses from large cohorts such as the CSPPT found that although cross‐sectional associations were observed, higher or lower B12 levels did not consistently predict incident diabetes during follow‐up (Liu et al. 2020). These discrepancies across studies may stem from differences in baseline nutritional status, genetic background, dietary patterns, and the prevalence of subclinical deficiencies in various populations. For example, recent studies have reported that FTO genotype may influence the association between the risk of different chronic diseases and dietary intake of micronutrients (Mohseni et al. 2025; Gholamalizadeh et al. 2023; Doaei et al. 2022). Considering gene variation, as well as methodological factors such as differences in study design, sample size, adjustment for confounders, and reliance on single baseline vitamin B12 measurements that may not reflect long‐term status, may contribute to the observed inconsistencies in findings.

The mechanism of the association between T2DM and vitamin B12 is not yet clear. A recent study indicated that serum B12 levels may be associated with glycemic fluctuations in T2DM (Li et al. 2022). One mechanism linking B‐complex vitamin (B6, B9, and B12) deficiency to type 2 diabetes primarily involves elevated homocysteine levels. Deficiency of these vitamins leads to homocysteine accumulation, which can impair pancreatic β‐cell function and increase insulin resistance (Mascolo and Vernì 2020). Additionally, elevated homocysteine promotes oxidative stress and inflammation, further disrupting glucose metabolism. These interconnected pathways provide a mechanistic explanation for how B‐vitamin deficiency may contribute to the development and progression of T2DM (Mursleen and Riaz 2017). Our findings demonstrate that vitamin B12 offers a greater protective effect for T2DM in those with the FTO TT genotype. This suggests the possibility of an interaction between genes and nutrients influencing disease risk, which lends credence to the growing field nutrigenomics. Notably, individuals carrying the AA and AT genotypes did not show any statistically significant association between consumption of vitamin B12 and T2DM. This is interesting considering that these genotypes are associated with greater obesity and metabolic risk (Doaei, Mosavi Jarrahi, et al. 2019; Yin et al. 2023). This may mean that for those who are genetically at risk for obesity, the FTO variant linked to metabolic dysregulation would blunt the possible positive effects of vitamin B12.

The practical uses for these findings are important. From a public health point of view, our findings indicate that a bioinformatics approach to T2DM with personalized dietary interventions using genetic information may improve prevention or management strategies. Particularly, individuals with the TT genotype of the FTO gene may benefit more from consuming higher levels of vitamin B12. Considering the increasing worldwide incidence of T2DM and its economic healthcare load (Abel et al. 2024; Gkrinia and Belančić 2025), studies of this nature seeking the interaction of genes with diet could help design precision nutrition strategies that enhance outcomes and optimize healthcare resources.

### 4.1 Study Limitations

This study has several limitations that should be considered when interpreting the findings. First, the case–control design limits causal inference. Second, dietary intake was assessed using an FFQ, which is subject to recall bias and measurement error. Although key confounders such as energy intake, physical activity, BMI, and carbohydrate intake were adjusted for, residual confounding cannot be ruled out. The absence of serum vitamin B12 measurements also prevents evaluation of the biological availability of dietary intake. In addition, the specific age range and demographic characteristics of the study population may limit generalizability. Some genotype subgroups (e.g., TT and AA/AT) included relatively small sample sizes, reducing statistical power and increasing the risk of type I or type II errors. The lack of HbA1c data and information on diabetes duration represents another limitation. Another limitation is the lack of detailed data on duration of diabetes and diabetes‐related microvascular complications. Detailed information on cardiovascular comorbidities and related medications (e.g., aspirin) was not systematically collected and may represent potential confounders. Although users of PPIs and vitamin B12 supplements were excluded, data on metformin use, including dose and duration, were not available; therefore, residual confounding due to metformin exposure cannot be ruled out. In addition, HbA1c values beyond diagnostic classification and duration of diabetes were not recorded, limiting assessment of long‐term glycemic control and disease severity. Finally, given the wide age range of participants (35–70 years), age could act as a potential confounder; this was addressed by adjusting for age as a continuous variable and performing sensitivity analyses across age strata. While the results suggest a potential protective effect of higher vitamin B12 intake in certain genotype groups, these findings should be interpreted with caution and validated in larger prospective studies.

Understanding better how vitamin B12 influences metabolism could lead to new strategies aimed at improving T2DM prevention. Such approaches could involve dietary changes, supplementation, or lifestyle changes that are precisely targeted based on an individual's genetics. There is an opportunity for advancement in healthcare with these personalized strategies. Future studies should try to confirm these results in bigger and more varied groups of people, and they should use longitudinal designs to figure out when and how things happen. In addition, studies that directly measure serum vitamin B12 and functional biomarkers like homocysteine and methylmalonic acid levels may give us a better understanding of the biological pathways at work. Looking into other gene‐nutrient interactions that include FTO and other micronutrients may help us understand even more how diet, genetics, and the risk of metabolic disease are all connected.

## 5 Conclusion

This study found that higher dietary vitamin B12 intake was associated with a lower risk of T2DM only among individuals carrying the TT genotype of the FTO rs9939609 polymorphism, whereas no significant association was observed in AT or AA carriers. These results underscore the potential role of gene–nutrient interactions in glucose metabolism and support the emerging concept of precision nutrition as a strategy for T2DM prevention.

## Author Contributions

Conceptualization: S.D., E.N.F., A.T., A.S.‐G., M.V., P.S., M.S. Formal analysis: S.D.; Methodology: M.G.H., A.M., M.A. Project administration: A.H.R., S.D., M.A., M.G.H. Writing – original draft: E.N.F., A.T., A.S.‐G. All authors read and approved the final manuscript.

## Funding

The National Nutrition and Food Technology Research Institute, Shahid Beheshti University of Medical Sciences, Tehran, Iran (Code 43015679) supported this study. The funding body had no role in the design of the study, and collection, analysis, and interpretation of data, or in writing the manuscript.

## Ethics Statement

The study protocol was approved by the ethics review committee of Shahid Beheshti University of Medical Sciences, Tehran, Iran (IR.SBMU.RETECH.REC.1404.299), and the research was carried out in accordance with the principles outlined. All participants voluntarily provided written informed consent.

## Consent

The authors have nothing to report.

## Conflicts of Interest

The authors declare no conflicts of interest.

## Data Availability

All data generated or analyzed during this study are included in this published article.
